# Molecular Epidemiology of Rifampicin Resistance in* Mycobacterium tuberculosis* Using the GeneXpert MTB/RIF Assay from a Rural Setting in India

**DOI:** 10.1155/2017/6738095

**Published:** 2017-10-26

**Authors:** Raghuprakash Reddy, Gerardo Alvarez-Uria

**Affiliations:** ^1^Department of Microbiology, Rural Development Trust Hospital, Bathalapalli, Andhra Pradesh, India; ^2^Department of Infectious Diseases, Rural Development Trust Hospital, Bathalapalli, Andhra Pradesh, India

## Abstract

The Xpert MTB/RIF assay can detect mutations in* rpoB* gene that confer rifampicin resistance (RR) using five overlapping probes (A, B, C, D, and E). In this study, we described our experience with the Xpert assay in a rural setting in India. During the study period, 3250 samples were processed. The result was unsuccessful in 5.7% of cases. For extrapulmonary specimens, the risk of unsuccessful result was higher in tissue biopsy and stool samples. Among samples positive for* Mycobacterium tuberculosis*, rifampicin resistance was indeterminate in 1.2% of them. Our results and a review of the literature showed that the most frequent mutations conferring RR were located in the region of Probe E (63.6%; 95% confidence interval [CI] 56.26–70.94), followed by Probe B (15.02%; 95% CI 11.94–18.10), Probe D (13.35%; 95% CI 10.01–16.69), Probe A (4.73%; 95% CI 1.92–7.54), and Probe C (1.61%; 95% CI 0.67–2.54). Although the high cost of the cartridges precluded using the Xpert assay for routine diagnosis of tuberculosis, our results demonstrate that the assay can be used to diagnose RR-tuberculosis in rural areas with limited laboratory infrastructure and could be a convenient tool to investigate the molecular epidemiology of RR in resource-limited settings.

## 1. Introduction

Rifampicin is arguably the most important drug in the treatment of tuberculosis (TB). Infection by rifampicin resistance (RR) TB requires long therapy with less effective and more toxic second-line drugs [1]. Proper treatment of RR-TB relies on prompt diagnosis [2]. However, diagnosis of RR-TB has been traditionally difficult, because it required sophisticated biosafety and laboratory infrastructures, which could be available in urban areas but hardly in rural settings.

The Xpert MTB/RIF assay is a WHO endorsed point-of-care molecular assay able to assess simultaneously diagnosis of TB and RR within two hours [3]. The assay requires minimal technical expertise and basic biosafety measures. The Xpert assay can detect mutations in five regions of the beta-subunit of the RNA polymerase enzyme* (rpoB)* gene using five overlapping probes (A, B, C, D, and E) [3]. Although the probe that confers RR is rarely reported in the clinical practice, the frequency of specific mutations in the* rpoB* region could provide useful information when studying the epidemiology of RR-TB in a particular region.

In this study, we describe our experience with the GeneXpert MTB/RIF assay and the molecular epidemiology of RR-TB in a rural setting in South India.

## 2. Methods

The study was performed in Anantapur, a district situated in the south border of Andhra Pradesh, India. In Anantapur, 72% of the population live in rural areas and 36% are illiterate [4]. Rural Development Trust General Hospital is a nonprofit 325-bed hospital in Bathalapalli, a rural village in Anantapur. The hospital belongs to a nongovernmental organization called Rural Development Trust.

We collected microbiological data from the Hospital Database of all samples processed with the GeneXpert MTB/RIF assay from 1 May 2011 to 31 December 2015. We also collected epidemiological data of patients who tested positive for RR. The assay was performed using the version G3 of the Xpert MTB/RIF assay until 7 April 2012 and the version G4 thereafter following manufacturer's instructions (Cepheid, Sunnyvale, CA, USA). The GeneXpert MTB/RIF uses molecular beacons in five overlapping regions of the* rpoB* DNA region. The probes are able to detect mutations in the codons 507 to 511 (Probe A), 511 to 518 (Probe B), 518 to 523 (Probe C), 523 to 529 (Probe D), and 529 to 533 (Probe E).

Unsuccessful results were classified in three groups according to manufacturer's instructions: “invalid” (failure of sample processing control because the sample was not properly processed or PCR was inhibited), “error” (failure of the probe check control because the reaction tube was filled improperly, because there was a reagent probe integrity problem, or because the maximum pressure limits were exceeded or there was a GeneXpert module failure) and “no result” (the test was stopped due to power outage) [5, 6]

A systematic review of the literature was performed. Both authors (RR and GAU) independently screened titles and abstracts from PubMed looking for studies describing mutations in the* rpoB* region using the GeneXpert MTB/RIF assay and published before 1 August 2017. We used the terms “Xpert” AND “mutations” AND (“rifampicin” OR “rifampin”) in PubMed. We excluded studies that did not mention the probes conferring RR.

Statistical analysis was performed using Stata Statistical Software (Stata Corporation, Release 14.2, College Station, Texas, USA). Confidence intervals (CI) for proportions were calculated using the Wilson method [7]. The pooled proportions were calculated using multilevel multinominal regression models with random intercepts for each study. The study was approved by the Ethics Committee of the Rural Development Trust Hospital.

## 3. Results

**Figure 1:**
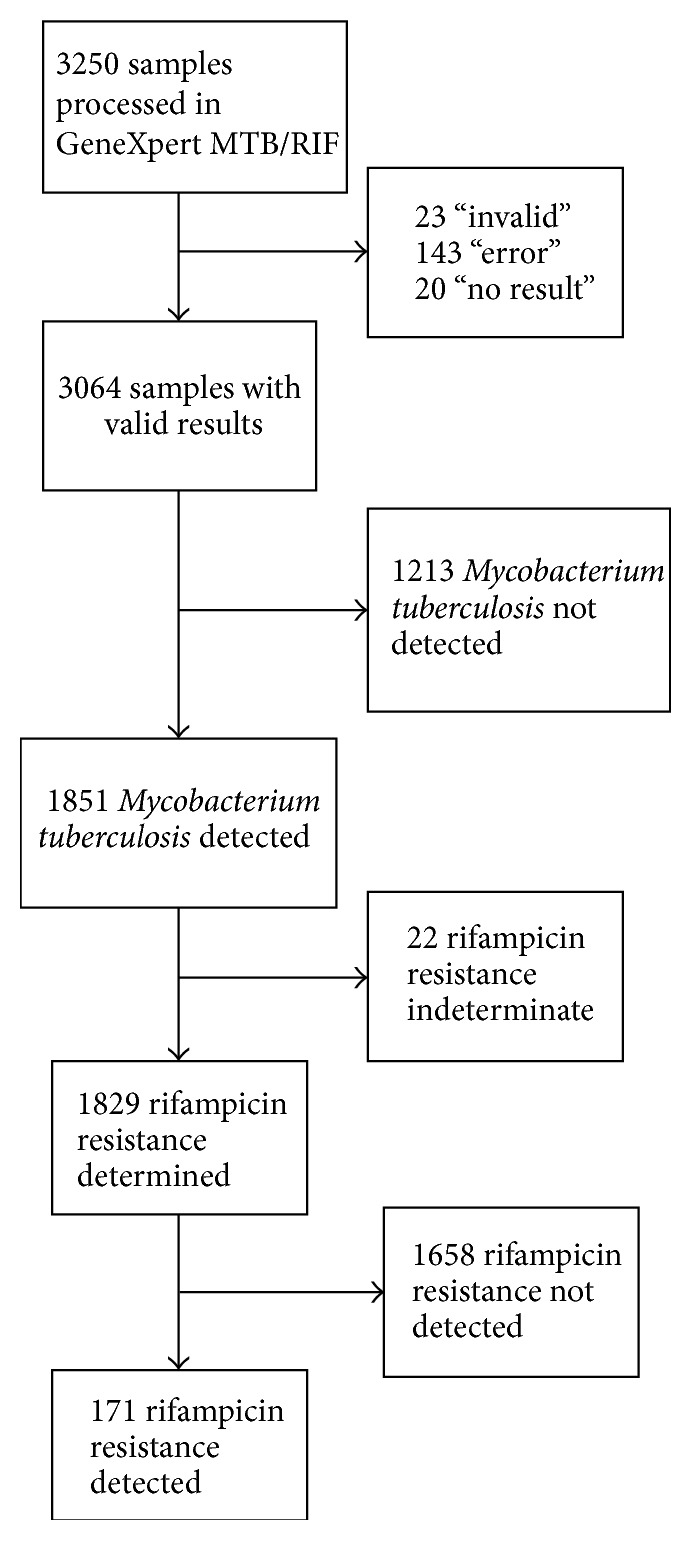
Flowchart of sample results using the GeneXpert MTB/RIF assay.

During the study period, 3250 samples were processed and the assay provided a valid result in 3064 (94.3%) cases and an unsuccessful result in 186 cases (5.7%) (Figure 1). Among unsuccessful results, 143 (76.9%) were classified as “error,” 23 (12.4%) as “invalid,” and 20 (10.7%) as “no result” due to power outages. The proportion of invalid results was 5.3% in sputum, 2.8% in pleural fluid, 3.2% in cerebrospinal fluid, 3.8% in ascitic fluid, 9.1% in pus, 19.1% in tissue biopsy, and 25% in stool. In a logistic regression model taking sputum specimens as the reference value, the odds ratio for having an unsuccessful result in extrapulmonary specimens was 0.52 (95% CI, 0.24–1.13) for pleural fluid, 0.58 (95% CI, 0.36–0.95) for cerebrospinal fluid, 0.7 (95% CI, 0.25–1.96) for ascitic fluid, 1.78 (95% CI, 0.9–3.53) for pus, 4.18 (95% CI, 1.38–12.69) for tissue biopsy, and 5.92 (95% CI, 1.57–22.27) for stool.

Out of 3064 valid results,* Mycobacterium tuberculosis* was detected in 1851 samples. Among samples positive for* Mycobacterium tuberculosis*, rifampicin resistance was indeterminate in 22 (1.2%), negative in 1658 (89.6%), and positive in 171 (9.2%).

Out of 171 patients diagnosed with RR-TB, 41 (24%) were female and the median age was 40 years (interquartile range, 31–49). RR-TB were detected in sputum (154), pus (5), cerebrospinal fluid (4), pleural fluid (4), pericardial fluid (1), synovial fluid (1), semen (1), and skin biopsy (1). The most common* rpoB* mutations were located in the region of Probe E (94, 55%), followed by Probe D (31, 18.1%), Probe B (26, 15.2%), Probe A (14, 8.2%), and Probe C (1, 0.6%). Combination of two probes was seen in five cases (2.9%): A&D in two cases, A&E in two cases, and B&D in one case.

**Figure 2:**
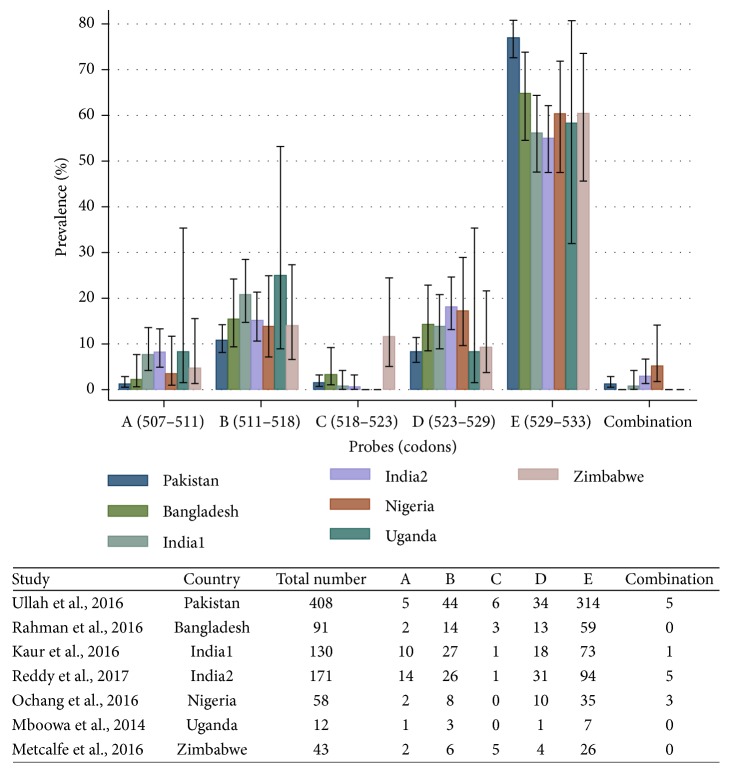
Comparison of the prevalence of probe failures conferring rifampicin resistance using the GeneXpert MTB/RIF assay in seven studies.

The PubMed search strategy showed 47 studies, and 41 were excluded because they did not provide information about the probes that conferred RR. Finally, six studies were included in the analysis [8–13]. The results of these studies and our study are compared in Figure 2. Probe E was the most common in all studies, followed by Probe B, although Probe D was more frequent than Probe B in our study and in the one by Ochang et al. [10]. Probe C was seen rarely, except in the study by Metcalfe et al. in Zimbabwe [13]. The pooled prevalence of probe mutations across studies was 4.73% (95% CI, 1.92–7.54) for Probe A, 15.02% (95% CI, 11.94–18.1) for Probe B, 1.61% (95% CI, 0.67–2.54) for Probe C, 13.35% (95% CI, 10.01–16.69) for Probe D, 63.6% (95% CI, 56.26–70.94) for Probe E, and 1.69% (95% CI, 0.73–2.66) for probe combinations. Overall, the most common combination of probes was B&D (6 cases), followed by A&B (2), A&D (2), A&E (2), D&E (1), and one triple combination of A&D&E.

## 4. Discussion

With 130,000 new cases in 2015, India had the highest population of RR-TB in the world [2]. Although two-thirds of the Indian population live in rural areas [4], diagnosis of RR-TB in rural settings is difficult because of the scarcity of qualified technicians and sophisticated laboratories. Our results demonstrate that the Xpert MTB/RIF assay can be used to diagnose RR-TB in rural settings with limited laboratory infrastructure.

In the present study, the proportion of successful results with the Xpert assay was 94.3%, which is higher than the one reported in studies from Botswana (85%) or India (92.8%) [6, 14]. As in previous studies, failure of the check control of the probe was the most common type of unsuccessful result, suggesting that improving the sample processing skills of the operators could reduce the proportion of failures [6, 14, 15]. Although the Xpert assay was designed for respiratory specimens, our study shows that the assay can provide valid results in extrapulmonary samples, such as pleural fluid, cerebrospinal fluid, and ascitic fluid. Our findings indicate the assay can also be used for other types of extrapulmonary specimens, such as pus, stool, or tissue biopsy, but the probability of unsuccessful results is higher.

In this study, the proportion of positive results for* Mycobacterium tuberculosis* was high. This can be explained by the fact that because of the relatively high cost of the cartridges, we stopped using the Xpert assay for the routine diagnosis of TB infection. The assay was mostly used to diagnose RR-TB in patients with acid fast bacilli (AFB) in sputum or as a rule-in test when extrapulmonary TB was suspected [16]. In the majority of patients who had AFB negative sputum and positive Xpert MTB, empirical treatment of TB was already initiated based on TB symptoms and radiographic findings [17].

Our results and the review of six studies performed in other developing countries from Africa and Asia indicate that the most common mutations conferring RR are located in the region of Probe E, followed by Probe B and Probe D, while mutations in the region of Probe A and Probe C were less common [8–13]. The predominance of Probe E and the fact that the Xpert assay does not provide information of specific mutations in the* rpoB* gene limit its value as an epidemiological tool to study RR-TB. However, we argue that other methods are more difficult to implement in resource-poor settings and data are readily available in countries that have incorporated the Xpert MTB/RIF assay in their National TB programme. Information about the probes conferring RR could be used to assess trends over time, identify pockets of transmission, or investigate outbreaks, especially when RR is secondary to mutations outside the Probe E region.

Previous studies in India have shown that the most common mutation conferring RR is located in the codon 531 of the* rpoB* gene (TCG→TTG), in which serine is substituted with lysine [18–20]. In these studies, other common* rpoB* mutations were located in the codon 516, which is in the Probe B region, and codon 526, which is in the Probe D region. Although in our study we did not determine specific* rpoB* mutations, the 531 codon is located in the Probe E region and was likely the predominant mutations in our patients.

The study has important limitations. We did not perform mycobacterial culture nor drug susceptibility tests. Thus, we could not estimate the proportion of false positive and negative results of the Xpert assay to diagnose TB or RR-TB compared with the gold standard. Moreover,* rpoB* gene sequencing was not done, so we could not establish the specific* rpoB* mutations nor, therefore, the specificity and sensibility of the assay to detect mutations in the* rpoB* gene [19]. In addition, information about previous episodes of TB-treatment was not available, so we could not determine the prevalence of RR-TB in patients with and without history of TB-treatment in the past.

## 5. Conclusion

In terms of access to healthcare, the rural population is usually underprivileged. In our setting, the high cost of the cartridges, compared with the standard AFB staining, precluded using the Xpert assay for the routine diagnosis of TB. However, the study showed that the Xpert MTB/RIF assay can provide valid results to diagnose RR-TB in a setting with limited laboratory infrastructure. Our results and a review of the literature indicate that the most common mutations that confer RR are located in the Probe E region, but there were substantial differences across studies. We argue that the Xpert assay can be a convenient, although limited, tool to investigate the molecular epidemiology of RR for National Tuberculosis Programmes in resource-poor settings.
